# Plasticity in the olfactory bulb of the maternal mouse is prevented by gestational stress

**DOI:** 10.1038/srep37615

**Published:** 2016-11-25

**Authors:** Laure Belnoue, Sarah Malvaut, Elodie Ladevèze, Djoher Nora Abrous, Muriel Koehl

**Affiliations:** 1 INSERM U1215, Magendie Neurocenter, Neurogenesis and Pathophysiology group, 146 rue Léo Saignat, Bordeaux-33077, France; 2 Université de Bordeaux, Bordeaux-33077, France

## Abstract

Maternal stress is associated with an altered mother-infant relationship that endangers offspring development, leading to emotional/behavioral problems. However, little research has investigated the stress-induced alterations of the maternal brain that could underlie such a disruption of mother-infant bonding. Olfactory cues play an extensive role in the coordination of mother-infant interactions, suggesting that motherhood may be associated to enhanced olfactory performances, and that this effect may be abolished by maternal stress. To test this hypothesis, we analyzed the impact of motherhood under normal conditions or after gestational stress on olfactory functions in C57BL/6 J mice. We report that gestational stress alters maternal behavior and prevents both mothers’ ability to discriminate pup odors and motherhood-induced enhancement in odor memory. We investigated adult bulbar neurogenesis as a potential mechanism of the enhanced olfactory function in mothers and found that motherhood was associated with an increased complexity of the dendritic tree of newborn neurons. This motherhood-evoked remodeling was totally prevented by gestational stress. Altogether, our results may thus provide insight into the neural changes that could contribute to altered maternal behavior in stressed mothers.

Motherhood, encompassing pregnancy and lactation, is a critical period associated with many neural and behavioral changes^1^,^2^. These changes include neuroendocrine, molecular and physiological adaptations that take place during pregnancy, parturition, and the post-partum period; they act in concert to reshape the brain and modify the behavior of the female producing a high level of maternal responsiveness along with changes in maternal mood, cognition, and stress regulation. In particular, towards the end of pregnancy and into lactation the response of the hypothalamo-pituitary-adrenal (HPA) axis to a variety of stressors is severely attenuated with a concomitant increase in basal corticosterone levels, adaptations that seem to be essential for the healthy development of the offspring^3^,^4^. Nevertheless, these changes come with a cost since reproduction is also a time when females become vulnerable to the effects of repeated or prolonged stress^5^,^6^,^7^,^8^,^9^, exhibiting in particular an alteration of maternal care^5^,^9^,^10^,^11^,^12^,^13^. However, the underlying mechanisms remain largely unknown.

Maternal behavior in rodents depends upon the detection of odorants and/or pheromones emanating from the pups and there is likely a need to form new olfactory memories for maternal behavior to be expressed. For example, i) postpartum female mice were found to retrieve young pups using the chemosensory cues that emanate from them without the use of visual or auditory clues^14^,^15^; ii) olfactory bulbectomy eliminates maternal behaviors^16^ and anosmia produced by nasal irrigation of zinc sulfate or depletion of noradrenaline within the main olfactory bulb results in a majority of female mice eating their offspring^17^,^18^; and iii) mothers lacking odor-evoked activity in the main olfactory epithelium show a deficit in pup retrieval^19^, further emphasizing the importance of chemoreception for maternal behaviors.

Neuroanatomically, the first site of olfactory information processing is the olfactory bulb, which is a site of continuous neurogenesis in rodents. This ongoing neurogenesis is involved in many aspects of olfactory function such as the memory processing of olfactory cues^20^, olfactory perceptual learning^21^, odor discrimination^22^, and short-term and long-term odor memory^23^,^24^,^25^,^26^,^27^,^28^,^29^. Furthermore, although the relationships between bulbar neurogenesis and maternal behavior have yet to be fully characterized, both nursing behavior and pup retrieval were found to be impaired after ablation of new neurons in the OB^30^,^31^.

Altogether this prompted us to hypothesize that motherhood may be associated to an improvement of olfactory functions linked to an enhanced plasticity in the OB and that gestational stress may alter this process. To test this hypothesis we analyzed the consequences of motherhood and gestational stress on several components of the olfactory function, including pup discrimination, non-biological odor discrimination and odor memory, and on both the number and the morphology of newborn granule neurons in the olfactory bulb.

## Results

### Impact of gestational stress on maternal behavior

**Figure 1:**
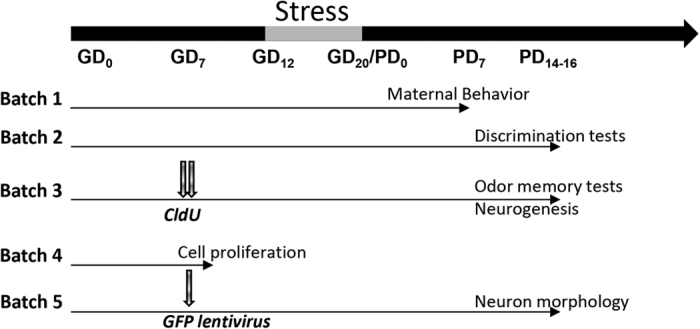
Schematic representation of the experimental design.

**Figure 2:**
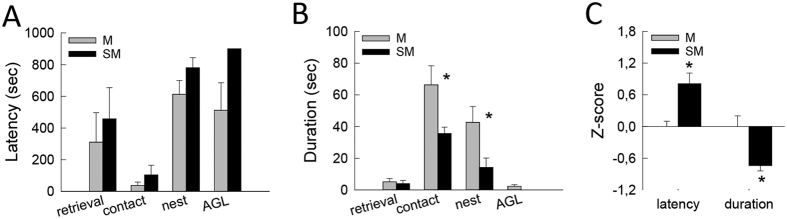
Impact of gestational stress on maternal behavior in the pup retrieval test. (**A**) Latency to first pup retrieval, pup contact, nest presence, and anogenital licking (AGL) in Mothers (M n = 6, grey symbols) and Stressed Mothers (SM n = 6, black symbols). (**B**) Duration spent in each behavior during the 15 min of the test. (**C**) Z-values for latency and duration of maternal behavior in Stressed Mothers compared to control Mothers (Zero value). *p < 0.01.

In a first step we verified that maternal behavior was impaired by gestational stress (Fig. 1, batch 1). Maternal behavior was examined by measuring latency (Fig. 2A) or duration (Fig. 2B) to initiate pup retrieval, to first contact pups, to settle down in the nest and to perform ano-genital licking. When each behavior was analyzed separately, only a decreased duration of pup contact (t_10_ = 2.25; p = 0.04) and nest presence (t_10_ = 2.32; p = 0.04) were observed in stressed mothers (SM) compared to mothers (M). In a subsequent analysis a z-normalization was applied^32^. For each behavior, a Z-score for latency and for duration was calculated as follows: Z = ((x-μ)/σ) where X represents the individual data for the observed parameter, and μ and σ the mean and standard deviation for the control group, respectively. Then a Z-latency score compiling all latencies (mean of each Z-latency) and a Z-duration score compiling all durations (mean of each z-duration) were calculated. These indicate how many standard deviations latency to engage in maternal behavior and duration of maternal behavior as a whole, i.e. pup retrieval, contact with pups, nest presence and ano-genital licking, differs between stressed mothers and control mothers. These analyses revealed that gestational stress significant increased latency (t_10_ = −2.71, p = 0.02) and decreased duration (t_10_ = 2.79, p = 0.01) of maternal behavior (Fig. 2C). Having shown that in our experimental conditions maternal care was impaired by gestational stress, we next analyzed whether the olfactory abilities of stressed mothers were altered, using odor discrimination and odor memory tasks.

### Impact of motherhood and gestational stress on pup discrimination

**Figure 3:**
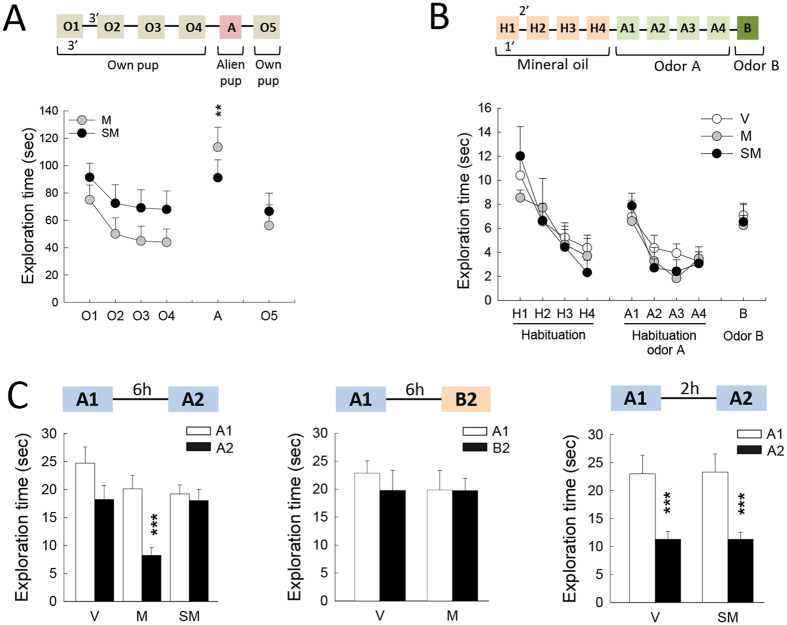
Impact of motherhood and gestational stress on odor processing. (**A**) Pup discrimination test in Mothers (M n = 10, grey symbols) and Stressed Mothers (SM n = 14, black symbols). Graph presents exploration time during the sequential exposures to own pup (O1 to O4, then O5) and alien pup (**A**) **p < 0.01 A ≠ O4 and A ≠ O5 in M group, NK test. (**B**) Odorant discrimination test in Virgins (V, n = 13, white symbols), Mothers (M n = 7, grey symbols) and Stressed Mothers (SM n = 8, black symbols). The graph represents the exploration time of a cotton swab impregnated with mineral oil (H1 to H4), eucalyptus (A1 to A4), and a mixture of eucalyptus (70%) and isoamylacetate (30%) (**B**). (**C**) Odor memory test. Left panel: Exploration time of a previously encountered odor after a 6 h delay in Virgins (V, n = 8), Mothers (M n = 7) and Stressed Mothers (SM n = 8) ***p < 0.001 A1 ≠ A2 in M group, NK test; middle panel: exploration time of a novel odor after a 6 h delay in Virgins (V, n = 8) and Mothers (M, n = 8); right panel: exploration time of a previously encountered odor after a 2 h delay in Virgins (V, n = 7) and Stressed Mothers (SM, n = 10) ***p < 0.001 A1 ≠ A2 in both V and SM groups, NK test.

We first evaluated the ability of mothers to discriminate their own pups from alien ones (Fig. 1, batch 2; Fig. 3A). When one of their own pup was presented, both mothers and stressed mothers did show a gradual habituation to its presence (O1 to O4: session effect: F_3,84_ = 4.27, p = 0.007; group effect: F_1,28_ = 2.50 p = 0.12; interaction: F_3,84_ = 0.08, p = 0.96). Then, when a pup belonging to another female was presented between two presentations of the dam own pups (O4-A-O5), exploration time for A was increased only in mothers (session effect: F_2,56_ = 13.81, p < 0.001; group effect: F_1,28_ = 0.06, p = 0.80; interaction: F_2,56_ = 3.04, p = 0.05; NK posthoc test in M group, A vs O4 and A vs O5 p < 0.01, in SM group, A vs O4 and A vs O5 p = 0.1 and 0.2, respectively), indicating that stressed mothers were unable to detect the foreign character of the pup.

### Impact of motherhood and gestational stress on odor discrimination

As these differences in pup recognition may be linked to changes in odor discrimination abilities, females of the three groups were subjected to a fine olfactory discrimination task using non-biological odors (Fig. 3B). Females were first habituated to a cotton-swab moistened with mineral oil (session H1 to H4: session effect: F_3,75_ = 19.61, p < 0.001; group effect: F_2,25_ = 0.05, p = 0.95; interaction: F_6,75_ = 0.99, p = 0.44). When odor A (Eucalyptus 10^−3^ M) was presented, all mice showed an initial increased in exploration time (H4 vs A1: session effect: F_1,25_ = 18.19, p < 0.001; group effect: F_2,25_ = 0.10, p = 0.90; interaction: F_2,25_ = 1.21, p = 0.32) and a subsequent decrease denoting habituation to the odor (session A1 to A4: session effect: F_3,75_ = 18.04, p < 0.001; group effect: F_2,25_ = 0.3, p = 0.75; interaction: F_6,75_ = 0.86, p = 0.53). Finally, when a dishabituation odor B quite similar to odor A (70% eucalyptus 10^−3^ M + 30% Isoamylacetate 10^−3^ M) was presented, again all the animals regardless of their group were able to detect its novelty (A4 vs B: session effect: F_1,25_ = 26.93, p < 0.001; group effect: F_2,25_ = 0.05, p = 0.95; interaction: F_2,25_ = 0.23, p = 0.8), indicating that neither motherhood nor maternal stress affects females’ ability to discriminate between two non-biological odors.

### Impact of motherhood and gestational stress on odor memory

The previous findings suggest that the alteration of pups recognition observed in stressed mother is not due to changes in odor discrimination but could be related to an alteration in olfactory memory processing (Fig. 1, batch 3). To test this hypothesis, we evaluated the ability of V, M, and SM groups to recognize an odor they encountered 6 h earlier, a delay at which most virgin mice do not recognize the odor (data not shown). As expected (Fig. 3C left panel), virgins displayed a similar amount of cotton swab sniffing during the 2 sessions of odor presentation, while mothers showed a decrease in sniffing time during the 2^nd^ presentation; stressed mothers showed a pattern of sniffing similar to that of virgins (session effect: F_1,21_ = 9.57, p < 0.01; group effect: F_2,21_ = 6.24, p < 0.01; interaction: F_2,21_ = 3.28, p = 0.05; NK post-hoc test: M group, p < 0.01). To ensure that the decrease in investigation time observed in mothers reflected odor recognition rather than nonspecific processes such as olfactory satiation or motivational problem, the specificity of odor recognition was assessed by exposing naïve virgins and mothers females to 2 different odors with a 6 h delay (Fig. 3C middle panel). Females from both groups showed a similar sniffing amount during the 1^st^ and the 2^nd^ exposure (session effect: F_1,15_ = 0.53, p = 0.47; group effect: F_1,15_ = 0.18, p = 0.67; interaction: F_1,15_ = 0.45, p = 0.51), confirming that the decreased sniffing time displayed when the same odor was presented twice was specific to odor recognition.

Finally, we tested whether the inability of stressed mothers to recognize a familiar odor after 6 h could be explained by a deficit in the processing of olfactory information. In such a case, these females would not be able to recognize an odor presented at a shorter delay, so we presented a new group of V and SM with the same odor with a 2 h delay (Fig. 3C right panel). Females from both groups spent less time sniffing the odor during the 2^nd^ presentation (session effect: F_1,15_ = 44.56, p < 0.0001; group effect: F_1,15_ = 0.002, p = 0.96; interaction: F_1,15_ = 0.006, p = 0.93) indicating that stressed mothers are capable of processing olfactory information.

### Impact of motherhood and gestational stress on neurogenesis in the olfactory bulb

**Figure 4:**
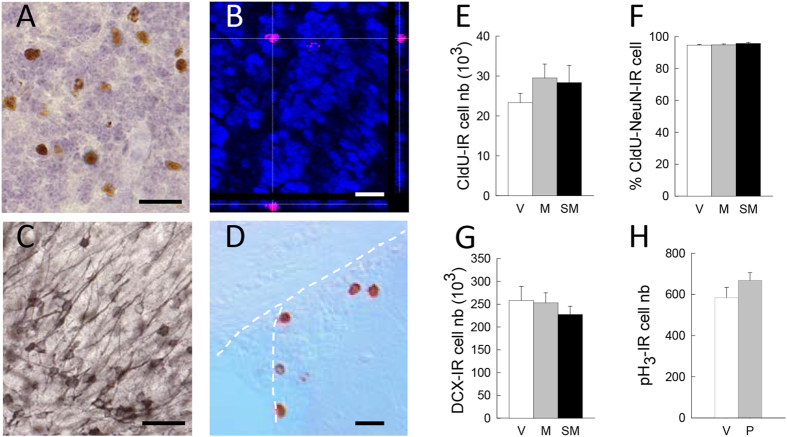
Impact of motherhood and gestational stress on adult neurogenesis in the olfactory bulb. Representative photomicrographs of (**A**) CldU-IR cells in a counterstained sagittal section of the olfactory bulb, (**B**) a colocalized CldU-IR (red staining) and NeuN-IR (blue staining) cell, (**C**) DCX-IR cells, and (**D**) pH3-IR cells in the SVZ. (**E**) Number of 4-wk-old CldU-IR cells in V (n = 7), M (n = 8) and SM (n = 9) mice, and (**F**) percentage of CldU-IR cells colocalized with the neuronal marker NeuN (n = 7 mice per group). (**G**) Number of DCX-IR cells in the olfactory bulb of V (n = 6), M (n = 5), and SM (n = 6) mice. (**H**) Number of proliferative pH_3_-IR cells in the SVZ of Virgin (V, n = 9) and Pregnant (P, n = 6) mice. Scale bar = 30 μm for (**A)** and (**C**), 20 μm for (**B**) and (**F**).

Given the role of adult neurogenesis in olfactory memory, we examined whether motherhood-induced enhancement in olfaction was associated to changes in the different steps of neurogenesis. As data from the literature reported an increase in neurogenesis in the OB of PD14 mothers^33^, we first analyzed the number of 4-week-old newborn neurons at this time point, when behavioral observations were made (Fig. 1, batch 3). To this end, females were injected with CldU at GD7. We found that neither motherhood, nor gestational stress modified the number of CldU-IR cells (Fig. 4A,E, F_2,21_ = 2.16, p = 0.14), or the percentage of neuronal differentiation (Fig. 4B,F, F_2,18_ = 0.3, p = 0.72). Consequently, the number of newborn neurons remained unchanged as well (F_2,18_ = 2.61, p = 0.10). We also measured the number of DCX-IR cells at the time of behavioral testing and confirmed the absence of modifications due to motherhood or stress (Fig. 4C,G, F_2,14_ = 0.93, p = 0.41). As these results do not corroborate data from the literature, we checked in our conditions whether cell proliferation was increased at the end of the 1^st^ week of gestation. A new batch of virgin and pregnant mice was sacrificed at GD7 and we analyzed the number of proliferating cells in the SVZ (Fig. 1, batch 4). We could not evidence any increase in cell proliferation in pregnant mice (Fig. 4D,H, t_13_ = −1.22, p = 0.24), which is in accordance with the lack of modifications in the number of 4-week-old CldU-IR cells.

**Figure 5:**
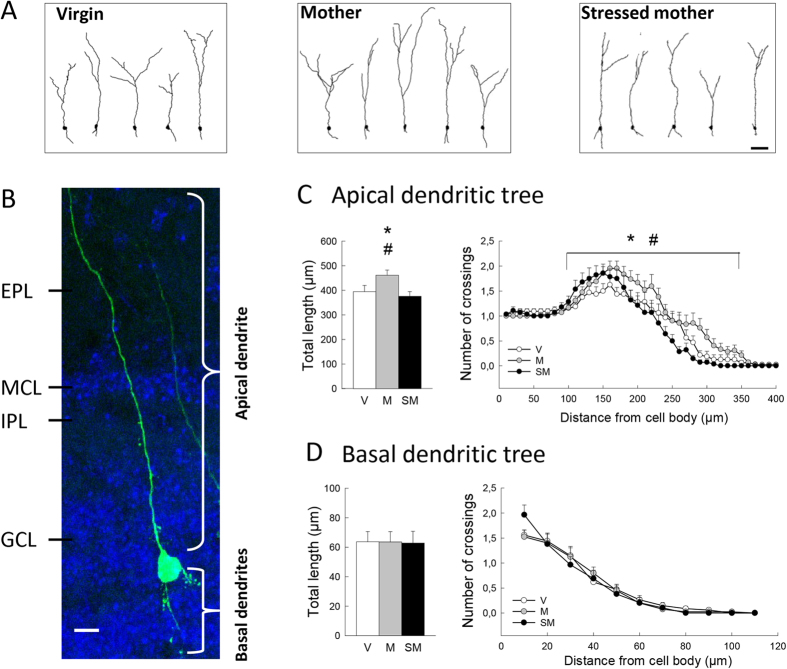
Impact of motherhood and gestational stress on the dendritic morphology of adult-born granule cells in the olfactory bulb. (**A**) Representative examples of the reconstruction of GFP expressing newborn neurons 4 weeks after virus injection for V (top panel, n = 7 mice, 32 neurons analyzed), M (middle panel, n = 6 mice, 42 neurons) and SM (bottom panel, n = 7 mice, 28 neurons). Scale bar is 50 μm. (**B**) Microphotograph of a newborn neuron expressing GFP. Scale bar is 10 μm. EPL = external plexiform layer; MCL = mitral cell layer; IPL = internal plexiform layer; GCL = granule cell layer. (**C,D**) Morphometric analyses of reconstructed neurons for apical (**C**) and basal dendrites (**D**): length and number of crossings as a function of distance from the cell body (Sholl analysis) were determined. Sholl analysis revealed an increased dendritic complexity of GFP+ neurons in the region between 100–350 μm from the cell body in Mothers compared to Virgins and Stressed Mothers. *p < 0.05 M vs V, ^#^p < 0.05 M vs SM groups, NK test.

As the relevance of adult neurogenesis also relies on synaptic integration of newborn cells, we then asked whether our behavioral modifications could be sustained by a modification in newborn neurons morphology. To do so we injected a GFP-lentivirus in the SVZ of both GD 7 pregnant mice and virgin mice (Fig. 1, batch 5). Mice were sacrificed at PD14 when neurons have integrated the granular zone of the OB (Fig. 5A,B). We found that the length of apical dendrites of 4 weeks old neurons was increased by motherhood and that this effect was blocked by gestational stress (Fig. 5C F_2,99_ = 4.28, p < 0.05; NK posthoc V group < M group and SM group < M group at p < 0.05). Similarly, motherhood increased dendritic complexity of newborn neurons in the region located 100–350 μm from the cell body, an effect absent in Stressed Mothers who did not differ from Virgins (Fig. 5C distance from soma effect: F_26,2574_ = 62.1, p < 0.001; group effect: F_2,99_ = 5.40, p < 0.01; interaction: F_52,2574_ = 1.44, p < 0.05; NK posthoc V group < M group and SM group < M group at p < 0.05). Neither motherhood nor stress impacted cell body area (V group = 68.42 + 1.43 μm^2^, M group = 65.48 + 1.41 μm^2^, SM group = 63.72 + 1.45 μm^2^; F_2,99_ = 2.39, p = 0.09) or the total number of nodes (F_2,99_ = 1.16, p = 0.31). These effects of motherhood and stress were specific of the apical dendritric tree as analysis of the basal dendrites did not reveal any differences between groups (Fig. 5D dendritic length, F_2,100_ = 0.003, p = 0.99; Sholl analysis distance effect: F_5,500_ = 90.70, p = < 0.001, group effect: F_2,100_ = 0.052, p = 0.99, interaction: F_10,500_ = 1.46, p = 0.14; nodes, F_2,100_ = 0.67, p = 0.51).

## Discussion

We report here that maternal stress disrupts maternal behavior and mothers’ capability to recognize their own pups, in addition of totally blocking the enhancement in odor memory induced by motherhood, indicating on one hand that motherhood improves odor processing and on the other hand that maternal stress interferes with the mechanisms involved in this improvement. Analysis of adult neurogenesis as an underlying process indicates that motherhood and maternal stress do not involve changes in the number of cells generated during gestation or the early postnatal period, but rather in their dendritic complexity. Altogether our results suggest that the behavioral consequences of motherhood and maternal stress may rely on a morphological remodeling of adult-born neurons in the olfactory bulb.

We found that gestational stress disturbed maternal behavior, a deficit which agrees with several studies showing that gestational stress reduces licking, grooming and nest building^9^,^10^,^11^. As olfaction is essential in mice to maintain an appropriate maternal behavior^34^, we compared olfactory abilities of non-stressed and stressed mothers. We found C57Bl/6 mothers able to discriminate their own pups from alien ones, which is in line with previous data^35^,^36^. Although this discrimination of a familiar vs unfamiliar pup does not imply that mice recognize the pups as their own, it contrasts with female rats’ behavior that lacks this specificity, and draws mice maternal behavior closer to ewes’^37^ and humans’ one^38^,^39^ that are capable of specific offspring recognition^34^.

We also report that motherhood is associated to an improved odor memory. To the best of our knowledge, this is the first time this type of memory is tested in relation to motherhood, and future studies will be needed to analyze the full consequences of this improvement in the context of maternal care. However, enhancement of other types of memory was previously reported in mothers, in particular their increased ability to learn and remember spatial environment, which is fundamental to their capacity to locate their pups, remember food location, and decreasing the probability for harmful encounters while away from the nest^40^. Altogether, the enhanced memory for odor observed in mothers is thus part of a general improved processing of the surroundings that seems central to successful parenting.

The analysis of stressed mothers’ behavior revealed an inability to distinguish between one of their own pups and an alien one, which could possibly reflect a deficit in odor processing. To test this hypothesis, females were first submitted to a discrimination task involving neutral odors, which revealed that virgins, mothers and stressed mothers were similarly capable of neutral odor discrimination. We then analyzed their odor memory performances and found that gestational stress totally abolished the beneficial effect of motherhood as stressed mothers were unable to restitute olfactory information with a 6 hours delay. When the delay was decreased to 2 hours, they performed as well as virgins, indicating that gestational stress may lead to deficits in odor memory consolidation or recall but spare memory trace encoding. Although this dataset indicates a deficit in odor processing, we cannot exclude the possibility that maternal stress also impairs the emotional value attributed to pup odors. Indeed, in both humans and rodents a shift in the valence attributed to infant odors was reported with motherhood, and recent mothers generally rate infant body odors more positively than non-mother female controls^34^,^38^.

Altogether, as olfactory abilities have been proposed as a necessary step for mother-infant bonding^34^, our results strongly suggest that the deficits registered in stressed mothers could underlie, at least in part, the altered maternal phenotype of stressed mothers.

Our next step was to look for a mechanism that could underlie the effects of maternal experience on odor processing. We focused on adult neurogenesis as previous data in mice have highlighted a strong link between odor processing and neurogenesis in the OB^22^,^25^,^26^,^27^,^29^,^41^. Moreover, there is some evidence linking motherhood to an increase in bulbar neurogenesis^31^,^33^, and several studies affecting more or less permanently neurogenesis have shown an impact on maternal behavior in mice^30^,^31^,^35^.

We first analyzed the number of newborn neurons generated during gestation, and could not evidence any difference neither in the number of cells generated at GD7, nor in the number of those cells that survived for 4 weeks (PD14 timepoint). We also did not find differences in the number of DCX-positive cells indicating that the number of cells generated during the last week of gestation and the first two weeks of life was not influenced by motherhood. Neuronal differentiation was not influenced either, and as a result bulbar neurogenesis, at least within the chosen time frame, remained unchanged following maternal experience. This result contradicts studies reporting a positive influence of motherhood on cell proliferation in the SVZ of pregnant female mice^31^,^33^,^42^. Although we cannot at the moment explain the exact origin of such a discrepancy, differences in strains or in thymidine analogue injections^43^,^44^ can be at play, along with other environmental differences such as the length of time females were exposed to males before and after plug detection^45^.

Altogether, in our conditions, behavioral and anatomical data indicate that bulbar adult neurogenesis, at least in term of cell numbers, is not linked to the olfactory memory modifications related to normal and disturbed gestation. Interestingly, these results are in accordance with a recent study showing that pup recognition is not linked to maternal bulbar neurogenesis^35^. However, pup recognition has been linked to paternal bulbar neurogenesis^46^, suggesting the existence of a parental sex-dependent link. Altogether, the sparseness of studies related to parental behavior and olfactory neurogenesis clearly highlights the crucial need for additional studies.

Nevertheless, because the effects of motherhood on neuronal plasticity are not only limited to changes in cell numbers, but extend to complex morphological changes such as adaptations in dendritic architecture, we next examined the impact of motherhood and stress on the dendritic arbor of newborn neurons. We found that motherhood is accompanied by a remodeling of 4-week-old newborn cells, characterized by a longer dendritic tree and an increased complexity of the apical dendrites. This result is in line with recent data showing an increase in the dendritic length of adult-born bulbar neurons in ewes interacting with lambs^47^ and an enhanced integration of bulbar neurons born in lactating mice^48^.

Taken together with our study, these results suggest that motherhood is accompanied by an enhanced synaptic integration of new neurons into the bulbar circuitry that is independent of species variations and total number of new OB neurons. Our data also reveal that gestational stress prevents the remodeling induced by motherhood since stressed mothers do not display any change in their dendritic arbor compared to virgin females. It is interesting to note that a similar effect has been highlighted in the striatal complex. Indeed, gestational stress was found to reduce dendritic complexity of Nucleus Accumbens neurons, and although the impact of motherhood was not studied *per se* in this study, it was suggested that gestational stress may prevent the enhancement in structural plasticity that normally occurs postpartum in this region^49^,^50^.

Undoubtedly, our results leave many questions unanswered. For example the mechanisms involved in motherhood-induced enhanced dendritic complexity of bulbar neurons and on the prevention of this effect in stressed mothers remain to be determined. Unfortunately, the role of the different factors known to impact dendritic development, such as cytoskeletal and transcriptional factors^51^,^52^, as well as sex and stress hormones^53^,^54^,^55^, has yet to be studied in the context of olfactory neurogenesis and dendritogenesis. Nevertheless, in the context of motherhood, it is likely that sex steroids, whose levels change at the time of parturition and during lactation, may be involved. Indeed, progesterone and estradiol, along with prolactin and oxytocin show variations that were found to modify maternal responsiveness^34^,^56^ and most of them were found to modulate dendritic growth^53^,^54^,^55^,^57^, albeit in systems other than the olfactory bulb. Another very likely candidate is GABA, one of the few factors known to modulate dendritogenesis in the OB. Indeed, GABA_A_ receptor mutant mice were found to exhibit impaired dendritic branching in OB granule cells suggesting that GABAergic transmission, which is increased in the OB at the time of parturition^58^, is important for proper dendritogenesis^59^.

Finally, because stress responsiveness is modified by pregnancy^60^,^61^, and because no study has yet fully analyzed the impact of gestational stress on mothers’ hormonal profile, one can only speculate about the mechanisms involved in the blockade of motherhood-induced dendritogenesis by gestational stress. Nevertheless, it is consistent with the reported role of glucocorticoids in other systems; for example, chronic treatment with glucocorticoids induces atrophy of apical dendrites on CA3 pyramidal cells^62^, and consistently, chronic restraint stress also reduces the length and branch points of apical dendrites on layer III of prefrontal cortex^63^. It is thus likely that an excess amount of glucocorticoids in stressed mothers may interfere with the dendritogenesis effect of motherhood, although this hypothesis and its underlying mechanisms remain to be studied.

To date, the functional implication of adult neurogenesis during the peripartum period is not fully elucidated. However, considering the functional importance of granule cells in odor representation^64^, and the link existing between maternal behavior and olfaction, our data strongly suggest that the remodeling of adult-born neurons observed in mothers may sustain the changes in olfactory processing occurring during the postpartum period. This hypothesis is corroborated by studies showing that adult-born granule cells are highly sensitive to sensory experience. In fact, the length and branching number of newborn granule cells are decreased after odor deprivation while they are increased in an odor-enriched environment^65^,^66^, a context also improving olfactory functions^25^. Hence, one can consider that the altered olfactory function and maternal behavior we observed in stressed mothers may be a consequence of a blockade in dendritic arbor growth that we observed in a context of undisturbed gestation. However, further studies will be needed to investigate this potential link.

Furthermore, although we have not tested this hypothesis here, it is possible that this remodeling may permanently distinguish virgins from mothers, which could contribute to the increased maternal responsiveness observed in multiparous mice compared to primiparous ones^18^, the so-called “maternal memory”^67^. Indeed it has been proposed that during the first parturition, the physiological factors and offspring cues required for maternal behavior combine to set up permanent alterations of its underlying pathways that would allow experienced mothers to display more rapid and efficient contact with their pups^34^. In line with this idea, reproductive experience was also found to have long-lasting and extensive consequences on non pup-related behaviors such as spatial learning and memory, and to imprint a permanent trace on the brain regions that regulate them, mainly the hippocampus^68^,^69^,^70^. Although the long-term remodeling of the olfactory system that we report here has yet to be analyzed, if its persistence is demonstrated, it may certainly contribute to “maternal memory”.

The damaging effects of gestational stress on offspring behavioral, physiological and neural development have been well documented but few studies have explored its effects on the mother, and most of them have focused on affective behaviors and their anatomical substrates. Thus, gestational stress was found to induce post-partum depression-like behavior^6^,^8^,^9^,^50^ or decreased behavioral flexibility^7^ in association to compromised structural plasticity in the hippocampus^69^,^71^, the median prefrontal cortex^7^ or the shell of the nucleus accumbens^50^. Gestational stress was also reported to affect neurogenesis in the DG of the hippocampus^4^,^72^. To the best of our knowledge, the present study is the first to investigate the impact of gestational stress on odor processing as a potential substrate of altered maternal behavior.

Although more work will be needed to fully elucidate these relationships, our study contributes to a much needed area of research exploring the effects of motherhood and maternal stress on the female brain, which ultimately should help improving the health and well-being of both mother and child, and also preventing the potentially devastating effects of early life stress.

## Materials and Methods

### Subjects

Three-month-old female C57Bl/6 J mice (Charles River, France) were housed under a 12 h light/12 h dark cycle (lights on from 8 pm to 8 am) in a temperature- (22 +/−3 °C) and humidity-controlled facility. Animals had *ad libitum* access to food and water. Females were initially group-housed to synchronize their ovarian cycle. Two to three weeks later, two groups were constituted; the first is a Virgin group (V) in which females had no physical contact with males. The second is a Mother group in which females were mated with C57Bl/6 J males; pregnancy was determined by the presence of a vaginal plug checked once a day at lights off. Upon plug detection (gestation day G0), females were removed from the breeding couples, and housed individually. Pregnant females were finally divided into two groups, a group of control Mothers (M) and a group of Stressed Mothers (SM). Everyday some virgin females were extracted from the collective cages and individually-housed in order to match housing conditions of Mothers. All mothers used in the study were primiparous.

All procedures involving animal experimentation and experimental protocols were approved by the Animal Care Committee of Bordeaux (CEEA50) and were conducted in accordance with the European community’s council directive of 24 November 1986 (86/609/ECC) that was running at the time the experiments were performed.

### Gestational stress

According to the model set up in our lab^73^, gestational stress was carried out from day 12 of gestation (GD12) until the end of pregnancy, occurring usually at gestation days 19 or 20 (GD19–GD20). Females were restrained in plastic transparent cylinders (50 mL Falcon tubes, 3 cm diameter, 11 cm long) under bright light for 45 min three times each day during the dark phase of the L:D cycle. Control mothers and virgins were left undisturbed throughout gestation. After delivery, dams and pups were left undisturbed until post-delivery day 7 (PD7) when maternal behavior was probed (batch 1) or post-delivery days 14 to 16 (PD14–PD16) when olfactory tests were performed.

### Maternal behavior

Maternal behavior was assessed during a pup retrieval test, a procedure that is often cited as an accurate marker of maternal care^13^ and widely used to assess maternal behavior. As previous work examining the temporal pattern of maternal behavior in rodents indicates that it declines significantly over the postpartum period, especially in conditions of separation such as that installed for the pup retrieval test^74^,^75^,^76^, we chose to perform this experiment on day 7 post-delivery (PD7), before levels of maternal behavior become too low to see a potential decrease after gestational stress (Fig. 1, batch 1). On the day of test, each dam was separated from her litter for 5 min during which pups were kept on a heating pad. Pups were reintroduced in the cage and randomly placed away from the nest (composed of a shredded nestlet); the mother was then reintroduced in the cage, and her behavior recorded during the 1^st^ fifteen min upon reunion. The following maternal behaviors were recorded: pup retrieval, pup contact (e.g. sniffing, licking), nest presence (being in physical contact with the pups or hovering in the location of the nest), and anogenital licking (AGL; the behavior consists of the mother visibly licking the hindquarters of the pups by firmly holding them between her front paws). All behaviors were recorded manually by a blind observer and scored for two parameters: duration and latency to initiate the behavior.

### Behavioral procedures to measure olfactory abilities

All behavioral procedures took place during the dark phase under red or very dim light. Pup discrimination and non-biological odor discrimination tasks were performed on the same animals on a random order on PD14 and PD16 (Fig. 1 batch 2). Odor memory task was performed on a separate cohort of females from the three experimental groups on PD14 (Fig. 1 batch 3), when new neurons born at GD7 have reach and integrated the OB^33^.

#### Pup discrimination

The ability of females to discriminate their own pups from alien pups (pups coming from a different litter) was assessed using a test based on habituation/dishabituation paradigm^35^,^36^. Only females from the M and SM groups were tested as females from the V group presented avoidance or aggressive behavior toward pups. Two females from the same experimental groups were tested simultaneously. Pups from the two litters were removed from the cage of their mother 15 min before the beginning of the procedure and put in a clean, heated cage, while mothers were transferred to the experimental room for habituation. One male pup from the tested mother’s litter (original litter) was introduced into the cage for 3 min sessions with a 3 min intersession delay (sessions O1 to O4). Then an alien pup was presented for 3 minutes (session A), and 3 min later, a pup belonging to the test female was presented (O5). All sessions were video-recorded and scored offline. The amount of time spent investigating the pup (sniffing or licking) was determined.

#### Odor discrimination

Mice ability to discriminate between two similar and closely related odorants was tested using again a habituation/dishabituation test^77^. Animals from the 3 groups were first exposed to a cotton-swab moistened with mineral oil (5 μl, *Sigma Aldrich*) and inserted through the rods of the mouse cage top, 10 cm above the cage floor, for four 60 s-sessions separated by a 120 s delay (sessions H1 to H4). Then a first odor (odor A: Eucalyptus 10^−3^ M, *Sigma Aldrich*) was presented 4 times (sessions A1 to A4). Finally a second odor (Odor B) corresponding to a mixture of Odor A and of 10^−3^ M Isoamylacetate (*Sigma Aldrich*) with a 70/30 ratio was presented for a 60 s-session (session B). For each mouse, time spent sniffing the cotton swab (snout 2 cm or less from the swab) was recorded and scored offline by a trained experimenter blind of the mice group.

#### Odor memory

Mice ability to remember a previously encountered odor was tested using the same experimental design: mice were presented with a cotton swab impregnated with 5 μL of odorant solution. A test session consisted of two 5 minutes odor presentations of the same odor (Odor A: Decanal caprinaldehyde 10^−3^ M, *Sigma Aldrich*) with different intervals. For each mouse, time spent sniffing the cotton swab (snout 2 cm or less from the swab) was recorded and scored by a trained experimenter. A significant decrease in investigation time during the second presentation indicates that mice recognize the previously-presented odor. In a pilot experiment using virgin C57Bl/6 J females, five intervals were tested and we found that females were able to recognize a previously encountered odor up to 4 h later, but that after 6 h this ability was lost in most individuals (data not shown). We thus selected a 6 h-delay to test the hypothesis that mothers would display an enhanced memory for odors. To assess the specificity of odor recognition, two control tests were subsequently performed on different batches of mice: different odors (Odor A and Odor B: cardamom, 10^−3^ M) were presented with a 6 h delay, or the same odor A was presented with a 2 h-delay.

### Chloro-2′desoxyuridine injections

On the 7^th^ day of gestation (GD7), all mice from batch 3 (Fig. 1) received two injections of 5-chloro-2′desoxyuridine (CldU, Sigma, 85.5 mg/kg/10 mL dissolved in saline solution) with a 10 hours delay and were sacrificed 4 weeks later.

### GFP-Lentivirus injections

A replication-deficient lentiviral vector was used to express eGFP in newborn granule cells of virgins, mothers, and stressed mothers. Mice from batch 5 (Fig. 1) were anesthetized with a mixture of ketamine/xylazine (80 mg/kg ketamine, 16 mg/kg xylazine) and received 100 μl of a local anesthetic (Lidocaïne) subcutaneously under the skin covering the skull. They received bilateral stereotactic injections of the eGFP-lentivirus in the subventricular zone (coordinates from Bregma: AP +0.8, ML+/−0.7, DV −2.4). The lentiviral particles were produced by transfection of 293 T cells with CMV-GFP, CMV-pGag/pol (viral proteins) and CMV-VSVg (envelop protein) plasmids. After 48 h, vectors were harvested and concentrated by ultracentrifugation to reach an average titer of 3.69 × 10^8 ^units/ml. Injections were performed using a Hamilton syringe to inject 1 microliter per site of infectious particles, at a rate of 10 nL/sec (with a 2 min wait before needle removal). This lentivirus infects both dividing and undividing cells independently of their phenotypes and allows cytoplasmic expression of eGFP, thus providing a tool for dendritic analysis of cells that reach the OB.

### Anatomical procedures

#### Immunohistochemistry

Mice from batches 3 and 5 were sacrificed on day 14 post-delivery, i.e. 4 weeks after CldU and GFP-lentivirus injections. Mice from batch 4 were sacrificed on gestational day 7 (Fig. 1). Mice were deeply anesthetized with pentobarbital (100 mg/kg) and perfused transcardially with 30 mL of PBS, pH = 7.3 and 30 mL of 4% paraformaldehyde in PB, pH = 7.3. Brains were dissected out of the skull and following a five-day postfixation period in paraformaldehyde, 40 μm sagittal (batch 3, neurogenesis at PD14) or coronal (batch 4, cell proliferation at GD7) and 60 μm coronal (batch 5, newborn neuron morphology) sections were cut on a vibratome and collected in PBS-azide (0.02%). For CldU immunohistochemistry an antigen retrieval step using 2 N HCl (30 min at 37 °C) was applied. For CldU, doublecortin (DCX), phosphorylated histone 3 (pH3), and GFP immunohistochemistries, one in ten free-floating sections were incubated with a rat monoclonal anti-CldU antibody (1/1000, Accurate), a rabbit polyclonal anti-DCX antibody (1/4000, Sigma), a rabbit polyclonal anti-pH3 antibody (1/1000, Millipore), or a rabbit monoclonal anti-GFP antibody (1/1000, Clontech), respectively. Then, sections were incubated with biotin-labeled goat anti-rat (1/1000, Amersham), or biotin-labeled goat anti-rabbit (1/500 for DCX and 1/200 for pH3 and GFP, Dako) secondary antibodies. Immunoreactivities were visualized by the biotin-streptavidin technique (ABC kit; Dako) with 3, 3′-diaminobenzidine (DAB) as chromogen for CldU, pH3, and GFP, and DAB-Nickel as chromogen for DCX. The phenotype of CldU-IR cells was examined by immunofluorescence labeling using a marker of mature neurons (NeuN, 1:1,000; Chemicon) revealed with an Cy5 goat anti-mouse (1/1000; Molecular Probe) and an Cy3 goat anti-rat (1/1000, Jackson) secondary antibodies.

#### Stereological analysis of staining

The number of immunoreactive cells was quantified in the left hemisphere under 1000x magnification with the optical fractionator method on a systematic random sampling of every tenth section along the temporo-median axis (CldU and DCX) or the septo-temporal axis (pH3), yielding to an average of 4 OB and 4 SVZ sections. We have previously shown that sampling of every fifth (which increases the number of sections) rather than every tenth section did not have any effect in the number of counted cells^22^. CldU-IR cells and DCX-IR cells were counted in the olfactory bulb using 50 × 50 μm and 30 × 30 μm counting frames, respectively, and at evenly spaced intervals of 300 × 300 μm, and 350 × 350 μm, respectively for CldU and DCX with an exclusion guard zone of 2 μm (Stereo Investigator software, MicroBrightField, VT, USA). All pH3-IR cells were counted in the left SVZ.

### Quantification of double-labeled CldU-NeuN cells

Double labeling was determined on 7 mice per experimental group using a confocal microscope (Leica DMR TCS SP2 AOBS) equipped with a 40x oil immersion objective, a Green225 Helium-Neon laser (543 nm), and a Red Helium-Neon laser (633 nm). Confocal acquisitions were performed with a 1.7 times zoom along the entire z-axis (25 μm) using 1 μm intervals, and were analyzed with the Metamorph software (Roper Scientific). Briefly, background was subtracted, a median filter was applied to gloss the images, and for each staining, images were overlaid, plane by plane, to determine colocalization of markers. For each animal, the number of CldU-IR cells analyzed corresponded to 25% of the total CldU-IR cells, as estimated with DAB staining, thus ranging from 200 to 500 cells.

### Morphometric analysis of GFP-labeled neurons

The morphometric analysis of virus-labeled neurons was performed with a X100 objective using a semiautomatic neuron tracing system (Neurolucida; MicroBrightField, Colchester, VT, USA). GFP-IR neurons were traced in their entirety, and area of cell body, number of dendritic nodes, and total dendritic length were calculated for both apical and basal dendrites. To measure the extent of dendritic growth away from the soma and the branching of dendrites at different distances from the soma, the concentric circle analysis of Sholl was carried out using the NeuroExplorer component of the Neurolucida program.

All the stereological and morphological analyses were performed by an experimenter blind of the mice group.

### Statistical analysis

For each experimental group, data were tested for normality using the Shapiro-Wilk normality test. Results are presented as mean + S.E.M. Data were analyzed using Statistica 8.0 (Statsoft) by ANOVAs followed by post hoc Newman-Keuls tests (NK test). For experiments comparing two groups, data were analyzed using a Student *t* test. Differences were considered significant when p < 0.05.

## Additional Information

**How to cite this article**: Belnoue, L. *et al*. Plasticity in the olfactory bulb of the maternal mouse is prevented by gestational stress. *Sci. Rep.* **6**, 37615; doi: 10.1038/srep37615 (2016).

**Publisher's note:** Springer Nature remains neutral with regard to jurisdictional claims in published maps and institutional affiliations.
